# Amid the possible causes of a very famous foxing: molecular and microscopic insight into Leonardo da Vinci's self‐portrait

**DOI:** 10.1111/1758-2229.12313

**Published:** 2015-08-19

**Authors:** Guadalupe Piñar, Hakim Tafer, Katja Sterflinger, Flavia Pinzari

**Affiliations:** ^1^ Department of BiotechnologyVienna Institute of Biotechnology (VIBT)University of Natural Resources and Life SciencesMuthgasse 11Vienna1190Austria; ^2^ Istituto Centrale per il Restauro e la Conservazione del Patrimonio Archivistico e Librario (ICRCPAL)Ministero per i Beni e le Attivita CulturaliVia Milano 76Rome00184Italy; ^3^ Present address: Consiglio per la ricerca in agricoltura e l'analisi dell'economia agrariaCentro di ricerca per lo studio delle relazioni tra pianta e suoloVia della Navicella 2‐4Rome00184Italy

## Abstract

Leonardo da Vinci's self‐portrait is affected by foxing spots. The portrait has no fungal or bacterial infections in place, but is contaminated with airborne spores and fungal material that could play a role in its disfigurement. The knowledge of the nature of the stains is of great concern because future conservation treatments should be derived from scientific investigations. The lack of reliable scientific data, due to the non‐culturability of the microorganisms inhabiting the portrait, prompted the investigation of the drawing using non‐invasive and micro‐invasive sampling, in combination with scanning electron microscope (SEM) imaging and molecular techniques. The fungus *E* *urotium halophilicum* was found in foxing spots using SEM analyses. Oxalates of fungal origin were also documented. Both findings are consistent with the hypothesis that tonophilic fungi germinate on paper metabolizing organic acids, oligosaccharides and proteic compounds, which react chemically with the material at a low water activity, forming brown products and oxidative reactions resulting in foxing spots. Additionally, molecular techniques enabled a screening of the fungi inhabiting the portrait and showed differences when different sampling techniques were employed. Swabs samples showed a high abundance of lichenized Ascomycota, while the membrane filters showed a dominance of *A* *cremonium* sp. colonizing the drawing.

## Introduction

**Figure 1:**
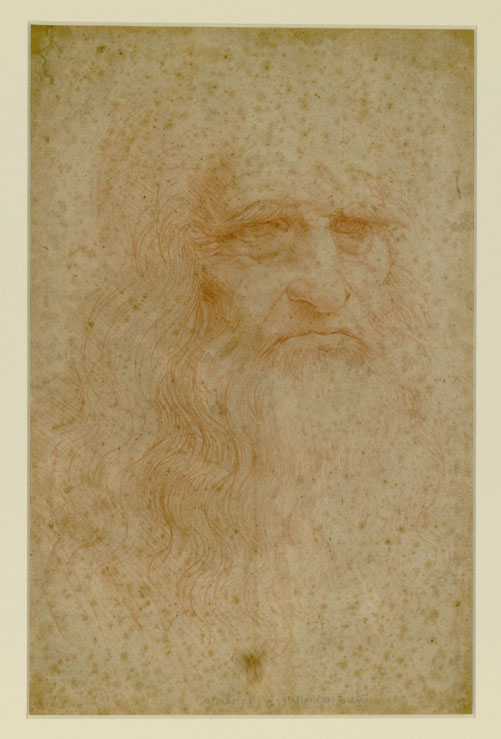
Leonardo da Vinci's self‐portrait drawn on paper with red chalk. The portrait was last exhibited in ‘Venaria Reale’ in 2012 in a clima‐box for 3 months and then brought to ICRCPAL for scientific analyses [Credits: Misiti, MC. (Central Institute for the Restoration of Archival and Library Heritage), ICRCPAL, Rome]. Picture by Corciulo, D. (ICRCPAL).

Leonardo da Vinci's famous self‐portrait was drawn in red chalk on paper. The drawing is housed at the Royal Library in Turin (‘Biblioteca Reale’, Italy) and is strongly affected by foxing spots (Fig. 1). The knowledge of the nature of the stains is a question of great concern because future conservation treatments and action taken to preserve the famous work should be founded on sound scientific data. Furthermore, study of the delicate object to date has not been received much attention from a biological point of view.

The dilemma of seeking to understand the dramatic phenomenon of the gradual fading of the portrait on the one hand, and of avoiding stress and further damage to the drawing on the other, has preoccupied our predecessors since the early 1950s.

A preliminary study performed by Gallo (1975) on the nature of the rust‐coloured stains affecting the drawing, based only on photographic images of the portrait dating back to 1952, 1962 and 1972, showed that by 1952, clear alterations comprising reddish‐brown colour spots of microbial origin were already visible on the edge of the sheet. Furthermore, the comparison of the photographic reproductions of the work showed no increase in the number of spots after 1952.

In a second report, Gallo (1986) reiterated that the reddish‐brown spots on the drawing were of microbial origin, but still being based solely on observation of the drawing by the naked eye. The further examination of the graphs produced between 1972 and 1987 by a thermo‐hygrograph placed in the closet where the portrait was kept, showed that the portrait was subjected to a Relative Humidity of 70–80% with peaks of 90%. Considering these results, Gallo (1987) informed the authorities about the necessity to disinfect the portrait with ethylene oxide. Nevertheless, in a later report Gallo and Plossi Zappalà (1987) hypothesized for the first time that the cause of the damage might have been of chemical origin.

The last report on the portrait was produced in 2005 (Valenti, 2005). For the first time, non‐invasive samplings were carried out on the drawing in order to evaluate the presence of viable microorganisms relating to the foxing spots. The sampling was performed with dry cotton swabs and the analyses based on classic cultivation on generic media (Sabouraud, MEA‐malt extract, CYA‐agar and Czapek yeast agar), resulting in no significant development of microorganisms. Contextually, a study on the presence of metals and chemical impurities in the drawing was carried out with portable X‐ray fluorescence spectroscopy (XRF). The absence of viable microorganisms and the presence of iron supported the hypothesis of a chemical cause in the formation of foxing spots on the portrait.

Finally, in 2012 the portrait was transferred from Turin to the ICRCPAL: ‘Istituto Centrale per il Restauro e la Conservazione del Patrimonio Archivistico e librario’, Rome, to be subjected to a thorough and complete series of investigations of its biological, chemical and physical properties, for the first time. A conference on the 2012 investigations was held in Rome, and some results were reported in the relevant proceedings (Misiti, 2014). The biochemical tests showed that at the time of sampling (2012), the portrait had no fungal or bacterial infections in place (Pinzari *et al*., 2014). As in previous attempts, the efforts to obtain a statistically significant number of colonies from the active microflora by cultivation techniques failed, although a wide range of culture media and isolation techniques were used in this case. The adenosine triphosphate (ATP) concentration values (measured according to Rakotonirainy *et al*., 2003) indicated a generic widespread contamination consistent with the deposition of atmospheric dust, naturally containing spores and biological materials. The analysis of the chitinase activity (analysis of 4‐Methylumbelliferyl N‐acetyl‐β‐D‐glucosaminide (4‐MUF‐NAG) according to Miller *et al*., 1998) detected the presence of fungal biomass in correspondence to the fluorescent areas of the drawing and the spots of foxing (often coinciding with the fluorescent zones).

With the aim of better assessing the current microbiological risk of the drawing, we performed a screening of the microbial community inhabiting the Leonardo da Vinci's self‐portrait based on non‐invasive and micro‐invasive sampling using diverse membrane filters and swabs, as well as micro‐tools (Figure S1), and a combination of scanning electron microscope (SEM) imaging and molecular biological techniques. DNA‐based techniques have enabled the identification of single fungal species in foxing on paper without the need for cultivation (Michaelsen *et al*., 2006). This fact represents an improvement because many candidate species that potentially may be associated with the spoiled material are non‐cultivable.

To this aim, we used a simple DNA extraction protocol, which allows the direct extraction of polymerase chain reaction (PCR)‐amplifiable DNA from very small samples of paper, swabs or membranes. The crude DNA extracts are used to amplify the fungal Internal transcribed spacer (ITS) regions, which are subsequently analysed by fingerprinting techniques, such as denaturing gradient gel electrophoresis (DGGE). In parallel, clone libraries containing the amplified fragments are constructed and screened by DGGE, allowing a band to clone comparison with the original DGGE fingerprints. Selected clones are sequenced and compared with the database to obtain a phylogenetic identification of the members of the microbial community. This molecular strategy can be combined with classical cultivation techniques, as well as microscopical and chemical analyses, to provide a comprehensive assessment of the state of the drawing.

## Results and discussion

### Stereomicroscope and SEM analyses

The spots of foxing observed under a microscope stereoscope showed surface erosion or thin filaments and cottony masses, especially on the verso of the work (Figures S1c and S2). These ‘objects’, wherever possible, were gently removed, in the presence of an expert conservator, and used for SEM observations.

**Figure 2:**
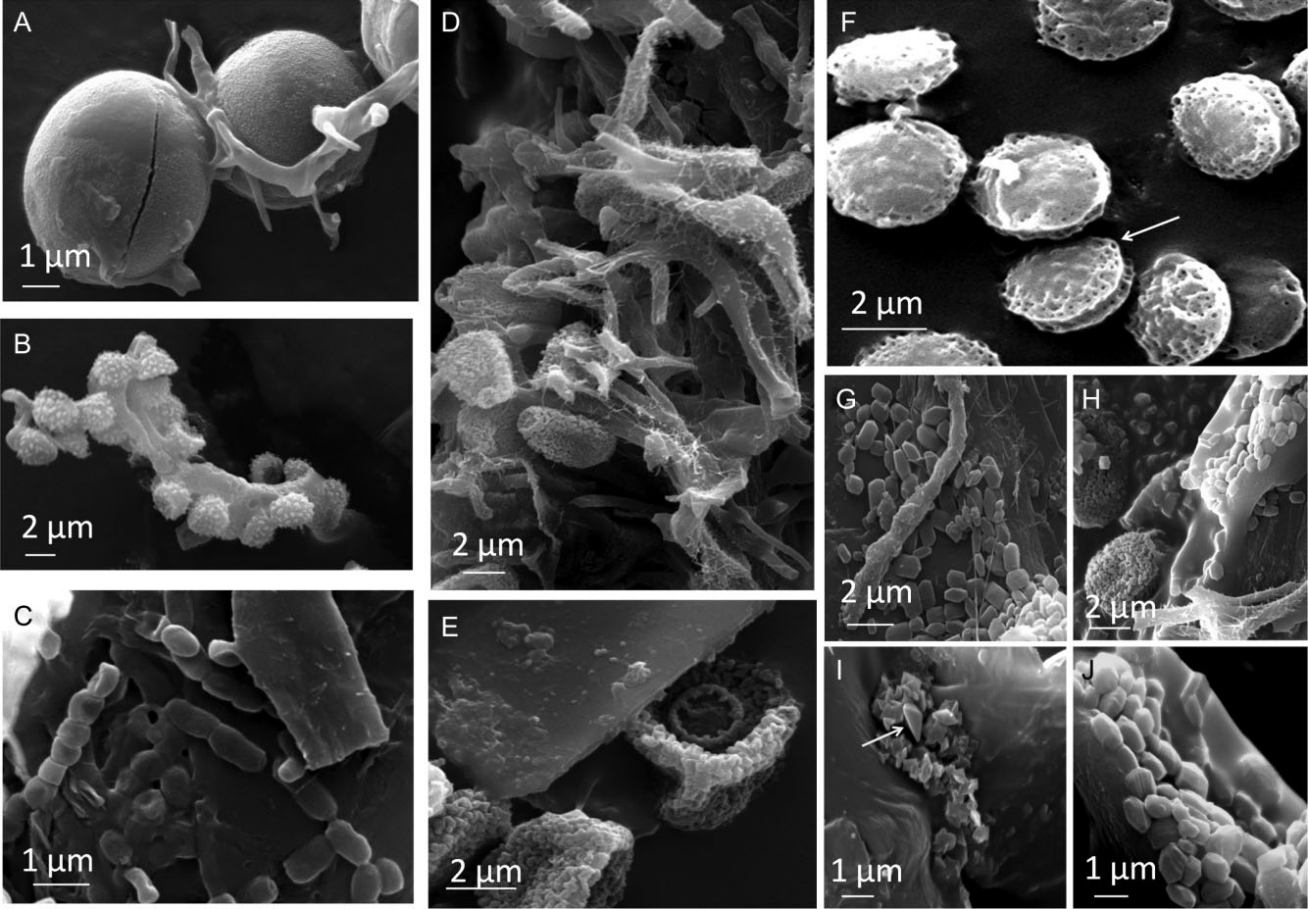
High vacuum, secondary electrons SEM images of fungal and bacterial structures. To obtain SEM imaging, micro‐samples were gently attached on stubs with carbon adhesive and analysed using a SEM instrument (EVO50, Carl‐Zeiss Electron Microscopy Group) equipped with a detector for secondary electrons (SE) at 20 keV. Samples were directly covered with gold with a Baltec Sputter Coater; the sputtering was performed under an Argon gas flow, at 50 mm working distance with 0.05 mbar of pressure and a current of 40 ma, for 60 s, to obtain a film of gold of about 15 nm. A. Globular structures (fungal conidia or algae) wrapped in filaments similar to fungal hyphae and characterized by the presence of longitudinal openings similar to germination seams. B. Clusters of echinated conidia not directly identifiable at the fungal genera level. C. Chain of spores of less than 1 μm in diameter, presumably belonging to filamentous bacterial structures (*Actinomycetales*), attached to a cellulose fibre taken from the drawing. D. Ellipsoidal conidia belonging to the fungal species *E. halophilicum*, collected from foxing spots on the verso of the drawing; the conidia present a significant variation in size (4–8/4–9 μm) that, in terms of shape, ornamentation and dimensions, are consistent with those of the anamorphous state of *E. halophilicum*, namely *Aspergillus halophilicu*s. E. Prominent scars in conidia were also pointed out. F. Lenticular, rough, with shallow furrow and bordered by low ridges; ascospores attributable to *E. halophilicum*. G–H. typical monoclinic prismatic monohydrate crystals of calcium oxalate attached to fungal structures and cellulose fibres sampled from the drawing. I. Typical tetragonal (arrow) dihydrate calcium oxalate crystals attached to cellulose fibres. J. Detail of prismatic monohydrate crystals of calcium oxalate embedded in a non‐defined matrix attached to the fungal structures, and cellulose fibres collected in foxing spots on the drawing.

SEM imaging showed that most of the material superimposed on the drawing contained several fungal spores, conidia and propagules of biological origin (Fig. 2A–E), like globular structures wrapped in filaments similar to fungal hyphae and characterized by the presence of longitudinal openings similar to germination seams (Fig. 2A), clusters of echinated conidia (Fig. 2B), and chains of spores of less than 1 μm in diameter, presumably belonging to *Actinomycetales* species (Fig. 2C). Several samples (70% of the spots analysed on the verso of the drawing) showed structures attributable to the fungal species *Eurotium halophilicum* Christensen, Papavizas & Benjamin (Christensen *et al*., 1959) all very collapsed and dried (Fig. 2D–F). Ellipsoidal conidia with a considerable variation in size (4–8 × 4–9 μm) were observed. The shape, ornamentation and dimensions of the documented conidia were consistent with those of the anamorphous state of *E. halophilicum*, namely *Aspergillus halophilicu*s Christensen, Papavizas & Benjamin 1961 (Samson and Lustgraaf, 1978). The typical ‘hairs’ on hyphae and lenticular, rough ascospores with shallow furrows, and bordered by low ridges, were identified by SEM observations (Fig. 2F). This fungus is an obligate xerophilic and osmophilic organism, with a high tolerance to water and salt stress and has the ability to develop in conditions that have a low content of substrates and nutrients (Zohri, 2000).

The occurrence of *E. halophilicum* has previously been reported in association with library material (Michaelsen *et al*., 2010; Montanari *et al*., 2012; Micheluz *et al*., 2015), and associated with foxing stains on paper by other authors (Gallo and Pasquariello, 1989; Florian and Manning, 2000). However, the last authors reported only the SEM image, and not the identification. The micro‐morphological characters of *E. halophilicum* found on the portrait were readily recognizable and perfectly matched the comparison with the fungal material already characterized by other authors (Montanari *et al*., 2012).

Regarding the role of fungi in the formation of foxing stains, it can be assumed that the efflorescences developed preferentially on cellulose fibres already undermined by a chemical attack (i.e. iron impurities and dust deposition) and were, therefore, the result of chemical foxing preceding the fungal attack. This hypothesis is consistent with the detection of higher concentrations of iron in the foxing spots than in the background (Bicchieri, 2014). However, it can be also hypothesized that fungi have developed following the onset of storage conditions without aeration and characterized by high relative humidity and high temperatures (Mosca Conte *et al*., 2014). These authors assert that the type of chromophores present in Leonardo's self‐portrait are similar to those found in ancient and modern paper samples aged in extremely humid conditions or within a closed environment.

In addition, the fungal role in the browning of paper could also be indirect and took place according to the mechanism described by Arai (1984; 1987). This author proposed that the formation of foxing does not depend on the vitality of the organisms, but on the compounds they leave between the cellulose fibres. These remains, rich in organic acids and metabolic products, may result, over time, in further browning due to melanoidines formed by the Maillard reaction (Arai, 1987; 2000; Arai *et al*., 1990). In such an eventuality, it is plausible that the metabolic products of *E. halophilicum*, capable of causing the foxing, remain on the drawing and continue to pose the threat of deterioration further to that already visible.

Associated with the paper fibres and fungal structures, typical calcium oxalate (CaOx) crystals were also documented (Fig. 2G–J) (Tazzolli and Domeneghetti, 1980; Pinzari *et al*., 2010). Extracellular oxalic acid (C_2_H_2_O_4_) has been demonstrated to play important roles in non‐enzymatic degradation of cellulose by fungi. Hastrup and colleagues (2011) proved that a 10 mM oxalic acid solution promoted a significant depolymerization effect on cotton cellulose. Pinzari and colleagues (2010) documented the biogenic formation of calcium oxalate crystals as a consequence of fungal activity and growth in vitro, on a range of CaCO_3_‐rich paper samples. Furthermore, the presence of non‐well‐identified ‘crystals’ on ancient paper manuscripts, and also in more recent library and archival materials, was described by some authors as associated with biodeteriorated materials (Gallo and Pasquariello, 1989; Montemartini Corte *et al*., 2003), thus suggesting that this phenomenon actually occurs on library materials when naturally spoiled by fungi that produce organic acids.

### Microbial screening: DNA extraction, amplification and DGGE fingerprinting

**Figure 3:**
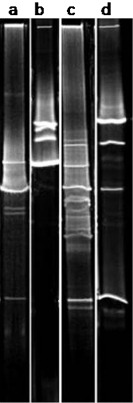
DGGE showing ITS1 amplified sequences from DNA isolated from cellulose nitrate membrane No. 2: lanes A–B, and from nylon membrane No. 1: lanes C–D. The DNA was extracted using the commercial fastDNA Spin kit for soil (MP Biomedicals, Illkrich, France), that incorporates a ribolysing step: lanes A, C or the method described by Principi and colleagues (2011), a freeze‐thawing based method: lanes B, D. The concentration and quality of the DNA extracts was assessed using a nanodrop ND‐1000 Spectrophotometer (peqlab Biotechnologie gmbh, Linz, Austria). Fragments corresponding to the ITS1, the ITS2 region and the 5.8S rRNA gene, were amplified with the primer pair ITS1 and ITS4 (White *et al*., 1990). For DGGE analysis, a nested PCR was performed with the PCR product of the first round as template DNA, using the primers ITS1GC with a 37‐base GC‐clamp attached to the 5′ end (Muyzer *et al*., 1993) and ITS2. All reactions were carried out as described by Michaelsen and colleagues (2006). DGGE gels were performed as previously described (Muyzer *et al*., 1993) using a D‐Code system (Bio‐Rad) in ×0.5 TAE (20 mm Tris, 10 mm acetate, 0.5 mm Na2 EDTA; ph7.8 with 8% (w/v) acrylamide). Gels were run at a constant temperature of 60°C with a voltage of 200 V over a period 5 h. The linear chemical gradient of denaturants used in this study (100% denaturing solution contains 7 M urea and 40% (v/v) formamide) was 30–50% denaturants.

The first step for the successful molecular analysis of the microbial community thriving on the Leonardo da Vinci's self‐portrait was the selection of an appropriate nucleic acid extraction method (Schabereiter‐Gurtner *et al*., 2001; Ettenauer *et al*., 2012). The efficiency of cell lysis, the quality and quantity of the extracted nucleic acid, its degree of purification and its size are crucial factors. Therefore, two different DNA extraction protocols – the one described by Principi and colleagues (2011) and the commercial FastDNa Spin kit for soil – were tested on membranes. Comparisons of DNA yield and purity, by NanoDrop measurements, indicated that the method employed by following the commercial extraction kit protocol renders a higher amount of DNA (4.4 and 4.06 ng DNA μl^−1^ for nylon and nitrate membranes respectively) than that based on the method described by Principi and colleagues (2011), which renders only 0.61 and 0.67 ng DNA μl^−1^ for nylon and nitrate membranes respectively. Furthermore, DGGE fingerprints of ITS‐amplified fragments (Fig. 3) revealed that the commercial kit method displayed a much higher microbial diversity, whereas the method described by Principi and colleagues (2011) showed that several DNA bands were missing. It can be concluded that the incorporated ribolysing step of the FastDNA Spin kit led to the complete lysis of all microbial cells. Comparing the different types of membranes tested (Fig. 3), the nylon membrane proved to be the most appropriate for use in combination with the selected DNA extraction kit. This type of membrane is more convenient to handle because of its smaller size, which allows for its direct introduction into the commercial tubes supplied by the extraction kit, thus eliminating the need for additional prior procedures that could introduce greater risk of contamination.

**Figure 4:**
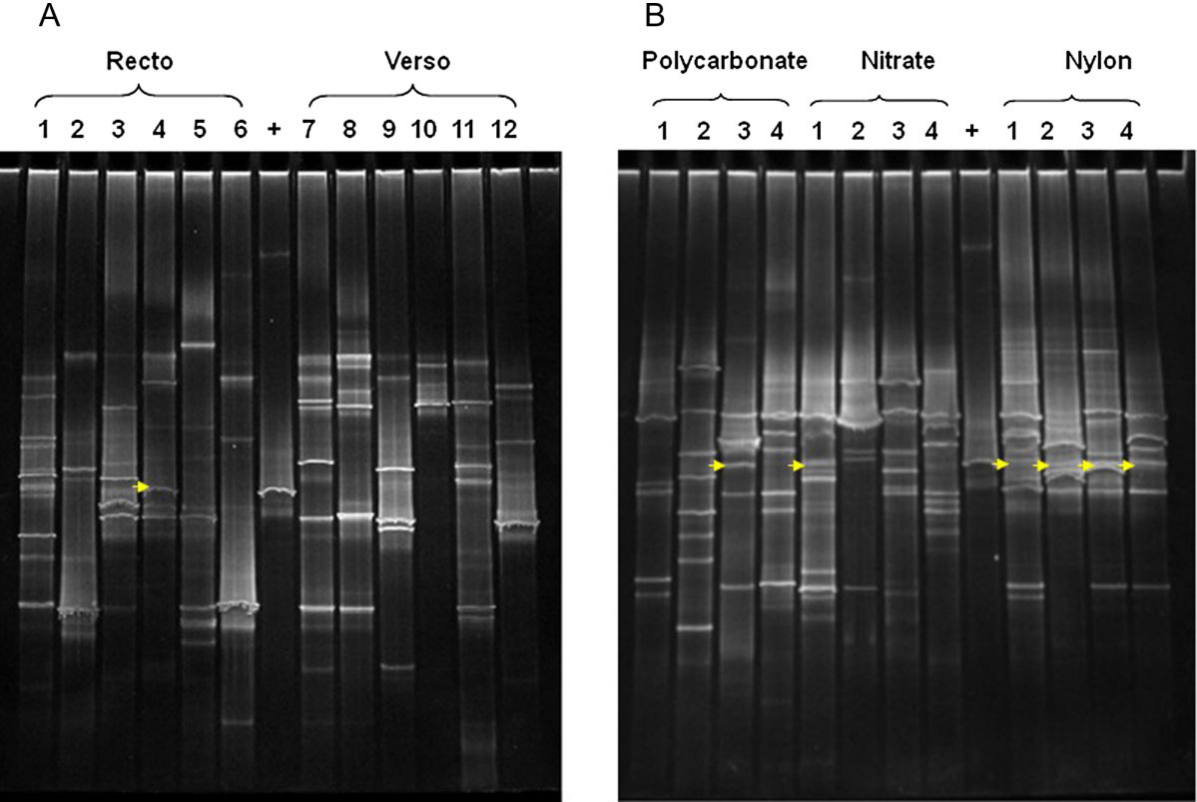
DGGE showing ITS1 amplified sequences from (A) cotton swabs samples (numbers indicate the number of the swabs) and (B) polycarbonate, cellulose nitrate and nylon membranes (numbers indicate the number of the membranes). The DNA of *E. halophilicum* was loaded as positive control (marked as **+**) and arrows indicate the position of the putative bands corresponding to *E. halophilicum* in the fingerprints. Conditions of PCR and DGGE as described for Figure 3.

Summarizing, the commercial kit used in this study once more proved to be the best DNA extraction method for very small sample amounts from a variety of substrates (Michaelsen *et al*., 2006; Ettenauer *et al*., 2012; Piñar *et al*., 2015a). Thus, the rest of the membranes, as well as the cotton swabs, were subjected to DNA extraction following this method. The DNA could be extracted from the 12 cotton swabs yielding a concentration of 3.76–7.18 ng DNA μl^−1^ and from all membranes yielding a concentration of 4.06–9.3 ng DNA μl^−1^. All extracted DNAs were further amplified by PCR with primers targeting the 16S rDNA of bacteria as well as the ITS regions of fungi. PCR analysis using bacterial‐specific primers yielded negative results for all samples. This may be due to a very low proportion of bacterial DNA in the total pool of extracted DNA (regarding that of fungi) or to its absence at all. Possible bias in the PCR reaction was excluded by the introduction of negative and positive controls. Nevertheless, for fungi, all PCR showed positive results, excluding the presence of PCR inhibitors in the extracted DNAs. DGGE fingerprints of ITS regions derived from the cotton swabs (Fig. 4A) were shown to be less complex than those derived from membranes (Fig. 4B), indicating a lower, only superficial, sampling recovery achieved from using swabs. The poor performance of swabs for sampling has been described by other authors (Moore and Griffith, 2007). Nevertheless, the fingerprints obtained from the membranes showed differences among each other, independently of the type used, indicating possible differences in the fungal communities in the different sampled areas.

As a positive control, the DNA of *E. halophilicum* was used (Fig. 4). The band corresponding to *E. halophilicum* could be seen to be present (just by visual inspection) in many of the fingerprints (Fig. 4). However, it must be stated that bands at the same position in the gel have the same melting behaviour, but not necessarily the same sequence (Muyzer *et al*., 1993). Only sequencing of bands can prove sequence identity. Unfortunately, in this study, attempts to cut and re‐amplify the presumable band corresponding to *E. halophilicum* failed.

### Phylogenetic identification of the mycoflora colonizing the portrait

To accomplish a detailed phylogenetic identification of the fungal communities seen on the Leonardo da Vinci's self‐portrait, four clone libraries containing the ITS fungal fragments were generated as follows: the DNA of three swabs from the recto (Nos. 1, 4 and 5) and three swabs from the verso (Nos. 8, 9 and 10) were pooled separately to obtain a single fungal clone library from each side (named CI for the recto and CII for the verso). From the membrane samples, those tested for the DNA extraction protocol (Nylon 1 and Nitrate 2) were further analysed by clone libraries as described in Table 1.

**Table 1:** Phylogenetic affinities of the ITS coding sequences detected in the Leonardo Da Vinci's drawing samples

Screened clones per sample %	Selected clone	Length (bp)	Closest identified phylogenetic relatives [EMBL accession numbers]	E‐values	Similarity (%)	Accession Numbers
Sample CI, swabs collected from the recto of the drawing						
59.%	CI‐K10	[560]	*Caloplaca dichroa* [EU563428].	0.0	99	KP828156
2.6%	CI‐K11	[596]	*Caloplaca austrocitrina* [EU563420].	0.0	99	KP828157
2.6%	CI‐K34	[551]	*Verrucaria nigrescens* [JX848570] from freshwater habitats in Europe.	0.0	92	KP828158
2.6%	CI‐K20	[578]	Uncultured *Stephanoascus* clone F9‐K2 [KC535141] detected in stuffing material from mummies in Palermo, Italy.	0.0	99	KP828159
			*Trichomonascus ciferrii* strain CBS 5295 [NR_111160].	1e‐118	85	
2.6%	CI‐K18	[583]	Uncultured fungus clone F3‐K10 [KC535113] detected in bones from mummies in Palermo, Italy.	0.0	100	KP828160
			*Gymnoascus petalosporus* [AB361639].	8e‐176	86	
12.8%	CI‐K2	[530]	Uncultured *Penidiella* clone F4‐K26 [KC535131] detected in hairs from mummies in Palermo, Italy.	0.0	99	KP828161
			*Penidiella venezuelensis* strain CBS 106.75 [EU019278].	0.0	94	
2.6%	CI‐K43	[575]	Uncultured *Phialosimplex* clones [KC535136, KC535127] detected in hairs from mummies in Palermo, Italy.	0.0	99	KP828162
			*Phialosimplex salinarum* strains [KF274685, KF274686, KF274692] isolated from a hypersaline habitat.	0.0	91	
2.6%	CI‐K30	[554]	Uncultured *Acremonium* clone AP3‐F4 [KF983520] detected in the Archimedes Palimpsest parchment book.	0.0	99	KP828163
			*Acremonium charticola* culture‐collection UOA/HCPF<GRC>:14413 [KC253940] common and emerging mould pathogens in Greece.	0.0	98	
2.6%	CI‐K36	[570]	Uncultured *Acremonium* clone F2‐K28 [KC535108] detected in clothes from mummies in Palermo, Italy.	0.0	99	KP828164
			*Acremonium* sp. CBS 115996 [EF042103].	0.0	96	
2.6%	CI‐K19	[527]	Uncultured ascomycete [AM901754] from indoor dust.	6e‐162	98	KP828165
			*Capnobotryella* spp. MA 4902 [AJ972847] isolated from Antalya/Turkey.	1e‐158	97	
2.6%	CI‐K1	[636]	Uncultured *Trebouxia*, photobiont from *Caloplaca* sp. s222 [JX036246].	5e‐178	93	KP828166
			*Trebouxia* sp. clade V LM‐2014 isolate L1401 [KJ754252].	3e‐176	93	
5.1%	CI‐K13	[707]	*Citrus sp.* [JN681150, JN661211].	0.0	99	KP828167
**Sample CII, swabs collected from the verso of the drawing**						
5.7%	CII‐K10	[601]	*Caloplaca austrocitrina* [EU563417, EU563419, EU563420].	0.0	99	KP828168
2.8%	CII‐K28	[597]	*Caloplaca dichroa* [EU563428].	0.0	99	KP828169
2.8%	CII‐K30	[541]	*Caloplaca aurantia* [AY233219].	0.0	99	KP828170
8.6%	CII‐K31	[588]	*Caloplaca dichroa* [EU563428].	0.0	97	KP828171
5.7%	CII‐K48	[598]	*Caloplaca dichroa* [EU563428].	0.0	97	KP828172
8.6%	CII‐K35	[578]	Uncultured *Stephanoascus* clone F9‐K27 [KC535144] detected in stuffing material from mummies in Palermo, Italy.	0.0	99	KP828173
			*Trichomonascus ciferrii* strain CBS 5295 [NR_111160].	1e‐118	85	
5.7%	CII‐K3	[578]	Uncultured *Stephanoascus* clone F9‐K49 [KC535146] detected in stuffing material from mummies in Palermo, Italy.	0.0	99	KP828174
			*Trichomonascus ciferrii* strain CBS 5295 [NR_111160].	7e‐117	85	
11.4%	CII‐K39	[600]	Uncultured fungal clones [KC535116, KC535114] detected in bones from mummies in Palermo, Italy.	0.0	99	KP828175
			*Gymnoascus* sp. NFCCI 2672 [JQ517292].	5e‐158	87	
11.4%	CII‐K47	[533]	Uncultured *Penidiella* clone F4‐K50 [KC535138] detected in hairs from mummies in Palermo, Italy.	0.0	99	KP828176
			*Penidiella venezuelensis* strain CBS 106.75 [EU019278].	0.0	99	
8.6%	CII‐K5	[576]	Uncultured *Phialosimplex* clones [KC535125, KC535140] detected in muscle and skin from mummies in Palermo, Italy.	0.0	99	KP828177
			*Phialosimplex chlamydosporus* strain UAMH 10961 [GQ169326] associated with infections in dogs.	0.0	91	
8.6%	CII‐K2	[576]	Uncultured fungus clone F4‐K2 [KC535126] detected in hairs from mummies in Palermo, Italy.	0.0	99	KP828178
			*Leptospora rubella* [HE774478].	0.0	92	
8.6%	CII‐K38	[570]	Uncultured fungus clone F4‐K28 [KC535132] detected in hairs from mummies in Palermo, Italy.	0.0	99	KP828179
			*Penicillium sp.* strain CBS 190.68 [JX841244].	0.0	89	
5.7%	CII‐K12	[520]	Uncultured fungus clone CMH052 [KF800143] fungal diversity in Kansas City indoor environments.	0.0	100	KP828180
5.7%	CII‐K40	[527]	*Cladosporium* sp. strains [NR_121333, NR_121332].	0.0	99	KP828181
**Sample NyI, nylon membrane**						
34.1%	NyI‐K5	[554]	Uncultured *Acremonium* clone AP3‐F4 [KF983520] detected in the Archimedes Palimpsest parchment book.	0.0	99	KP828182
			*Acremonium charticola* culture‐collection UOA/HCPF<GRC>:14413 [KC253940] common and emerging mould pathogens in Greece.	0.0	98	
2.4%	NyI‐K1	[554]	Uncultured *Acremonium* clone AP3‐F4 [KF983520] detected in the Archimedes Palimpsest parchment book.	0.0	99	KP828183
			*Acremonium charticola* culture‐collection UOA/HCPF<GRC>:14413 [KC253940] common and emerging mould pathogens in Greece.	0.0	97	
2.4%	NyI‐K13	[554]	Uncultured *Acremonium* clone AP3‐F4 [KF983520] detected in the Archimedes Palimpsest parchment book.	0.0	99	KP828184
			*Acremonium charticola* culture‐collection UOA/HCPF<GRC>:14413 [KC253940] common and emerging mould pathogens in Greece.	0.0	97	
4.9%	NyI‐K23	[540]	Uncultured *Acremonium* clone F2‐K5 [KC535100] detected in clothes from mummies in Palermo, Italy.	0.0	100	KP828185
			*Acremonium nepalense* isolate Ppf33 [GU586837].	0.0	94	
2.4%	NyI‐K7	[570]	Uncultured *Acremonium* clone F2‐K28 [KC535108] detected in clothes from mummies in Palermo, Italy.	0.0	99	KP828186
			*Acremonium* sp. CBS 115996 [EF042103].	0.0	96	
2.4%	NyI‐K18	[570]	Uncultured *Acremonium* clone F2‐K28 [KC535108] detected in clothes from mummies in Palermo, Italy.	0.0	99	KP828187
			*Acremonium* sp. CBS 115996 [EF042103].	0.0	96	
14.8%	NyI‐K35	[574]	Uncultured fungus clone CMH376 [KF800467], fungal diversity in Kansas City indoor environments.	0.0	99	KP828188
2.4%	NyI‐K17	[573]	Uncultured *Phialosimplex* clones [KC535127, KC535120, KC535119] detected in hairs and muscle from mummies in Palermo, Italy.	0.0	99	KP828189
			*Phialosimplex chlamydosporus* strain UAMH 10961 [GQ169326] associated with infections in dogs.	0.0	91	
2.4%	NyI‐K43	[533]	Uncultured *Penidiella* clone F4‐K50 [KC535138] detected in hairs from mummies in Palermo, Italy.	0.0	100	KP828190
			*Penidiella venezuelensis* strain CBS 106.75 [EU019278].	0.0	99	
2.4%	NyI‐K4	[576]	Uncultured fungus clone F4‐K2 [KC535126] detected in hairs from mummies in Palermo, Italy.	0.0	99	KP828191
			*Leptospora rubella* [HE774478].	0.0	92	
2.4%	NyI‐K30	[576]	Uncultured fungus clone F4‐K2 [KC535126] detected in hairs from mummies in Palermo, Italy.	0.0	99	KP828192
			*Leptospora rubella* [HE774478].	0.0	92	
2.4%	NyI‐K6	[570]	Uncultured fungus clone F4‐K28 [KC535132] detected in hairs from mummies in Palermo, Italy.	0.0	99	KP828193
			*Penicillium ornatum s*train CBS 190.68 [JX841244].	0.0	89	
2.4%	NyI‐K16	[602]	Uncultured *Rhizopus* clone F4‐K34 [KC535134] detected in hairs from mummies in Palermo, Italy.	0.0	99	KP828194
			*Rhizopus oryzae* strains [KJ417572, AY803930], human pathogenic species.	0.0	99	
7.4%	NyI‐K29	[604]	Uncultured *Rhizopus* clone F4‐K34 [KC535134] detected in hairs from mummies in Palermo, Italy.	0.0	100	KP828195
			*Rhizopus oryzae* strains [KJ417572, AY803930], human pathogenic species.	0.0	99	
2.4%	NyI‐K2	[562]	*Penicillium chrysogenum* strains [KJ413371, KC009826, JQ422603, JN561259].	0.0	99	KP828196
4.9%	NyI‐K25	[563]	*Penicillium chrysogenum* strains [KJ413371, KC009826, JQ422603, JN561259].	0.0	99	KP828197
7.4%	NyI‐K45	[520]	*Cryptococcus laurentii* strains [JQ247577, JQ247574].	0.0	99	KP828198
**Sample N2, cellulose nitrate membrane**						
91.5%	N2‐K2	[554]	Uncultured *Acremonium* clone AP3‐F4 [KF983520] detected in the Archimedes Palimpsest parchment book.	0.0	99	KP828199
			*Acremonium charticola* culture‐collection UOA/HCPF<GRC>:14413 [KC253940] common and emerging mould pathogens in Greece.	0.0	98	
6.4%	N2‐K4	[557]	Uncultured *Acremonium* clone F2‐K5 [KC535100] detected in clothes from mummies in Palermo, Italy.	0.0	99	KP828200
			*Acremonium nepalense* isolate Ppf33 [GU586837].	8e‐171	95	
2.1%	N2‐K13	[562]	Uncultured *Acremonium* clone F2‐K5 [KC535100] detected in clothes from mummies in Palermo, Italy.	0.0	99	KP828201
			*Acremonium nepalense* isolate Ppf33 [GU586837].	8e‐171	95	

Clone libraries containing the ITS fungal regions (using the primers ITS1/ITS4; White *et al*., 1990) were performed as described by Piñar and colleagues (2015a). A total of 50 clones from each clone library were screened in a DGGE gel as described by Schabereiter‐Gurtner and colleagues (2001). Selected clones were externally sequenced by Sanger sequencing with a fleet of 16 ABI 3730xl (GATC Biotech, Germany). Comparative sequence analysis was performed by comparing pair‐wise insert sequences with those available in the public online database NCBI, using the blast search program (Altschul *et al*., 1997). The resulting sequences of the fungal clones have been deposited at the GenBank: Genetic sequence database at the National Center for Biotechnical Information (NCBI) and the corresponding accession numbers are given in this table.

### Results derived from cotton swabs

Curiously, results derived from cotton swabs showed that most of the screened clones were related to lichenized Ascomycota (see Table 1, Sample CI and Sample CII), as *Caloplaca* spp. (Vondrák *et al*., 2009), detected at both the recto (61.6% of clones) and verso of the portrait (25.7% of clones), as well as *Verrucaria nigrescens* (Orange, 2013), only detected at the recto (2.6% of screened clones). The reason of finding these fungi may be related to the abundance of their spores in the air and their superficial deposition on the drawing, as they were found in a higher proportion on the recto of the portrait. The portrait was not conserved or transported with sterile procedures, and species of both *Caloplaca* and *Verrucaria* are widespread in Italian regions and common in Turin, where the portrait was conserved before the analysis (Nimis, 1993).

Other sequences that could be found at both the recto and verso of the drawing (see Table 1, Sample CI vs Sample CII) were sequences most related (99–100% similarity) to uncultured clones, as: uncultured *Stephanoascus* sp., uncultured fungal clones detected in bones, uncultured *Penidiella* sp. and uncultured *Phialosimplex* sp. (the relative abundance of these clone is given in Table 1, Sample CI and Sample CII), all of which sequences having previously been detected in deteriorated mummies located in Palermo (Piñar *et al*., 2013). In addition, sequences most related to *Phialosimplex* sp. have been also found in deteriorated parchment (Piñar *et al*., 2015a). This fungus seems to have a strong proteolytic activity, and it has been described as a pathogen of animals and humans (Sigler *et al*., 2010). Nevertheless, recent studies have reported on the isolation of fungal strains most related to *Phialosimplex* sp. from hypersaline habitats, i.e. marine environments (Ravindran *et al*., 2012), and a salt mine (Greiner *et al*., 2014). In the last study, the isolated strains were established as a new species, namely *Phialosimplex salinarum*, and all strains were shown to degrade cellulose and proteins (Greiner *et al*., 2014).

Sequences derived from cotton swabs that could be detected only on the recto of the drawing (Table 1, Sample CI) were: two clones (2.6% each) affiliated with uncultured *Acremonium* clones previously detected in an old parchment book (Piñar *et al*., 2015b) and in biodeteriorated human remains (Piñar *et al*., 2013) respectively. One additional clone (2.6%) affiliated with an uncultured ascomycete from indoor dust (Pitkäranta *et al*., 2008). Finally, 7.7% of the sequenced clones were shown to be affiliated with DNA of plants, being 2.6% associated with *Trebouxia* sp. (Muggia *et al*., 2014), which is the photobiont from the detected *Caloplaca*, and 5.1% with *Citrus* sp. The ITS primers ITS1 and ITS4, even if widely used for the specific amplification of fungi, are known to amplify plant ITS regions as well (White *et al*., 1990).

Additionally, sequences derived from cotton swabs that could be detected only on the verso of the drawing (Table 1, Sample CII) were: two clones (representing each 8.6% of the sequences) related to uncultured fungal clones previously detected in the hairs of deteriorated mummies located in Palermo (Piñar *et al*., 2013). The rest of the sequences (5.7% each) were affiliated with an uncultured clone detected in indoor environments (Rittenour *et al*., 2014) and with cultivated species of *Cladosporium*.

### Results derived from membranes

The sequences obtained from membrane samples were shown to differ partially from those derived from swab samples. The most significant difference was that those sequences related to lichens, dominant in the clone libraries of swab samples, were not retrieved from the clone libraries of membrane samples (Table 1, Sample NyI and Sample N2). Instead, sequences most related to uncultured *Acremonium* clones (Piñar *et al*., 2013; 2015b) were shown to be dominant (48.6% and 100% of clones screened from the nylon and nitrate membranes respectively). These sequences were also detected in the clone library CI (cotton swabs of the recto of the drawing; Table 1, Sample CI).

Nevertheless, the fungal diversity displayed by the nylon membrane proved to be much higher than that of the nitrate membrane (see Table 1, Sample NyI vs Sample N2; and the rarefaction curves shown in Figure S3) and, in addition to the *Acremonium*‐related sequences, 14.6% of the sequences proved to be affiliated with an uncultured clone detected in indoor environments (Rittenour *et al*., 2014). In addition, 2.4% of the sequences were affiliated with uncultured *Phialosimplex* sp. (Piñar *et al*., 2013), also detected in the clone libraries CI and CII (see Table 1, Sample CI and Sample CII). Four additional clones (2.4% each) were most affiliated with uncultured fungal clones detected in hairs from mummies. All of these last sequences were also detected in the clone library CII (verso of the drawing; Table 1, Sample CII). Finally, the remaining clones retrieved from the nylon membrane were shown to be most related to uncultured and cultivated species of *Rhyzopus* (9.7%), as well as to cultivated species of *Penicillium* (9.7%) and *Cryptococcus* (7.3%), with none of these sequences being detected in any of the others clone libraries. Both detected *Acremonium* and *Penicillium* species have been associated with foxing stains and paper discolouration in previous studies (Gallo and Pasquariello, 1989).

It is worthy of mention that no clone related to *E. halophilicum* could be detected in the clone libraries performed with the swabs and membranes. This fact could be attributed to the low relative abundance of the DNA of this fungus in the total pool of extracted DNA (Muyzer *et al*., 1993) or to the partial degradation effects on its DNA due to the natural ageing of conidia.

### Conclusions

The results obtained in this study clearly show that the use of membranes, especially the nylon membranes, is a good alternative for non‐invasive sampling of objects of art (Cappitelli *et al*., 2010), as well as a more accurate method for further fingerprinting and phylogenetic analyses. In contrary, the use of cotton swabs results in an overestimation of the surface microbiota, not really involved in the deterioration phenomenon.

The commercial DNA extraction kit used in this study displayed a much higher microbial diversity than the method described by Principi and colleagues (2011) and, once more, proved to be the best DNA extraction method for very small sample amounts, minimizing the risk of contamination and representing a relatively quick DNA extraction procedure.

Although no clone related to *E. halophilicum* could be retrieved from the clone libraries, the fungus was recognized by SEM imaging. This fungus is strongly xerophilic and oligotrophic and grows preferentially in absence of ventilation, consistently with the storage conditions under which the portrait was stored between 1929 and 1930.

The role of *E. halophilicum*, and possibly of other fungi such as *Phialosimplex, Penicillium* and *Acremonium* species, in the browning of the paper was indirect and possibly took place (and thus, it is still taking place) according to the phenomena described by Arai (2000, and references therein), which do not depend on the vitality of the organisms, but on the remaining compounds that fungi leave on cellulose fibres after death.

The direct role of fungi in cellulose oxidation and the formation of foxing stains could be attributed to the production of oxalic acid, taking into account the presence of crystals of calcium oxalate and surface erosion connected to some foxing spots. The presence of fungal material, oxalates and other biological compounds undoubtedly represents a very real threat to the conservation of the drawing because the browning phenomenon could be caused by slow ongoing chemical reactions that are independent of the viability of the detected microorganisms, and the current conditions of preservation.

## Supporting information


**Fig. S1.** Sampling. The samples obtained from the drawing were all documented and ‘mapped’ on a coordinate grid, plotted on a scale reproduction of the work. **a, d)** Series of cotton swabs were rubbed gently on the foxed areas on the recto: Swab 1, Swab 2, Swab 3, Swab 4, Swab 5 and Swab 6, and on the verso of the drawing: Swab 7, Swab 8, Swab 9, Swab 10, Swab 11 and Swab 12. **b, e)** Three types of membranes were used: cellulose nitrate membranes (filter pore size: Millipore 12:45 n.13906‐47‐BUN), marked as: Nitrate 1, Nitrate 2, Nitrate 3, and Nitrate 4; nylon membranes (nylon Whatman filters pore size: 0.45 μm, diameter 47 mm, Cat No. 7404‐004), marked as: Nylon 1, Nylon 2, Nylon 3 and Nylon 4) and polycarbonate membranes (Whatman Nucleopore polycarbonate), marked as: Poly 1, Poly 2, Poly 3 and Poly 4, all used on the recto of the drawing. **c)** In addition, a series of samples was taken using micro‐tweezers, with the aid of a stereomicroscope (Leica MZ16) (Pinzari *et al*., 2010). Micro‐particles, filaments, individual raised fibres and ‘objects’ in contact with the work were collected, particularly those relating to the spots of foxing on the verso of the drawing. The ‘objects’ were fixed on stub sample holders for observation under a scanning electron microscope [*Credits*: Misiti, MC. / Central Institute for the Restoration of Archival and Library Heritage, ICRCPAL, Rome]. Picture by Corciulo, D. (ICRCPAL).

**Fig. S2.** Detail of the surface of the drawing. At the recto: **a, b, c;** and at the verso: **d, e, f.** Pictures obtained with a digital camera connected to a Leica MZ16 stereoscopic microscope fitted with low temperature fibre optic lighting. Micro‐particles, filaments, individual raised fibres and ‘objects’ in contact with the work, particularly in relation to the spots of foxing, are visible. The rust traits visible in picture **a** and **b** are the red chalk traits.

**Fig. S3.** Rarefaction curves done with R and the VEGAN package. The number of expected species (*y*‐axis) is plotted as a function of the number of samples (*x*‐axis). Blue line: sample CI; black line: sample CII; green line: sample Ny1 and red line: sample N2.
