# Supplementary figures and images for: Amid the possible causes of a very famous foxing: molecular and microscopic insight into Leonardo da Vinci's self‐portrait
Source: Environ Microbiol Rep. 2015 Aug 19;7(6):849–59. doi: 10.1111/1758-2229.12313 (PMC4959533; doi:10.1111/1758-2229.12313)

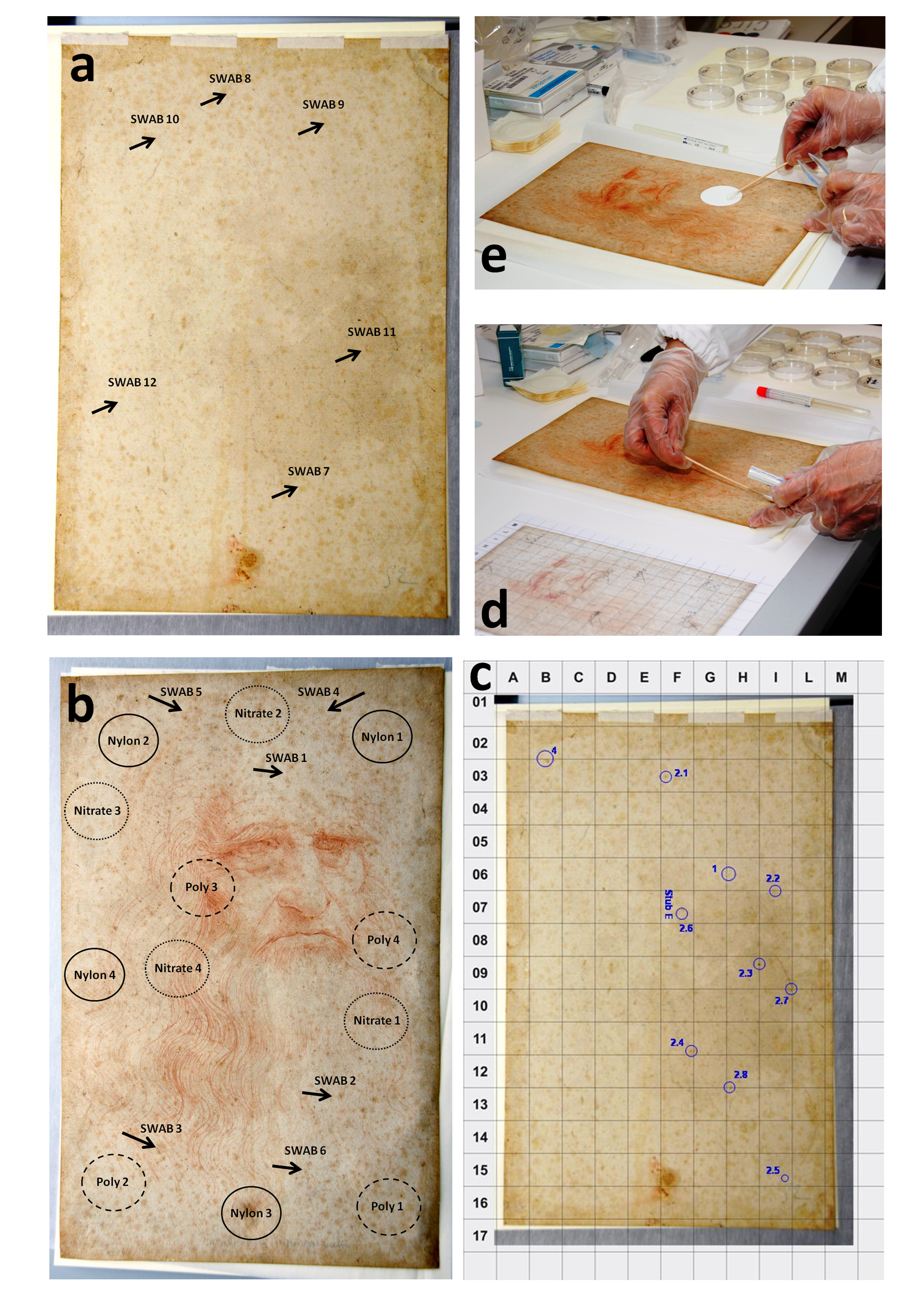

Supplement: Supplementary file 1 — Fig. S1. Sampling. The samples obtained from the drawing were all documented and ‘mapped’ on a coordinate grid, plotted on a scale reproduction of the work. a, d) Series of cotton swabs were rubbed gently on the foxed areas on the recto: Swab 1, Swab 2, Swab 3, Swab 4, Swab 5 and Swab 6, and on the verso of the drawing: Swab 7, Swab 8, Swab 9, Swab 10, Swab 11 and Swab 12. b, e) Three types of membranes were used: cellulose nitrate membranes (filter pore size: Millipore 12:45 n.13906‐47‐BUN), marked as: Nitrate 1, Nitrate 2, Nitrate 3, and Nitrate 4; nylon membranes (nylon Whatman filters pore size: 0.45 μm, diameter 47 mm, Cat No. 7404‐004), marked as: Nylon 1, Nylon 2, Nylon 3 and Nylon 4) and polycarbonate membranes (Whatman Nucleopore polycarbonate), marked as: Poly 1, Poly 2, Poly 3 and Poly 4, all used on the recto of the drawing. c) In addition, a series of samples was taken using micro‐tweezers, with the aid of a stereomicroscope (Leica MZ16) (Pinzari et al., 2010). Micro‐particles, filaments, individual raised fibres and ‘objects’ in contact with the work were collected, particularly those relating to the spots of foxing on the verso of the drawing. The ‘objects’ were fixed on stub sample holders for observation under a scanning electron microscope [Credits: Misiti, MC. / Central Institute for the Restoration of Archival and Library Heritage, ICRCPAL, Rome]. Picture by Corciulo, D. (ICRCPAL). [file EMI4-7-849-s001.tif]

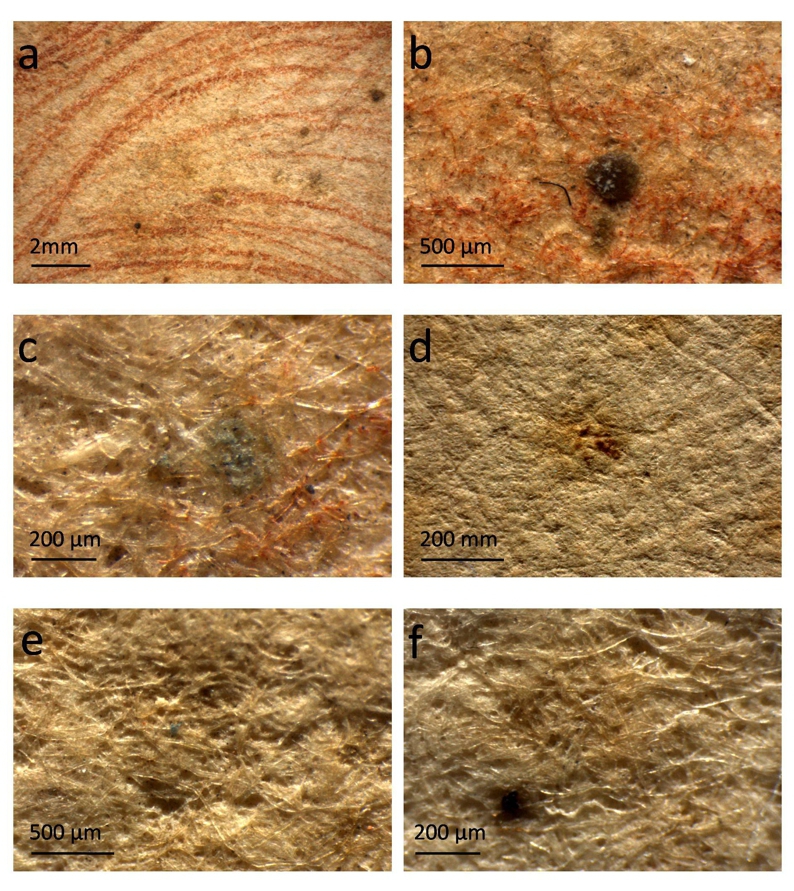

Supplement: Supplementary file 2 — Fig. S2. Detail of the surface of the drawing. At the recto: a, b, c; and at the verso: d, e, f. Pictures obtained with a digital camera connected to a Leica MZ16 stereoscopic microscope fitted with low temperature fibre optic lighting. Micro‐particles, filaments, individual raised fibres and ‘objects’ in contact with the work, particularly in relation to the spots of foxing, are visible. The rust traits visible in picture a and b are the red chalk traits. [file EMI4-7-849-s002.jpg]

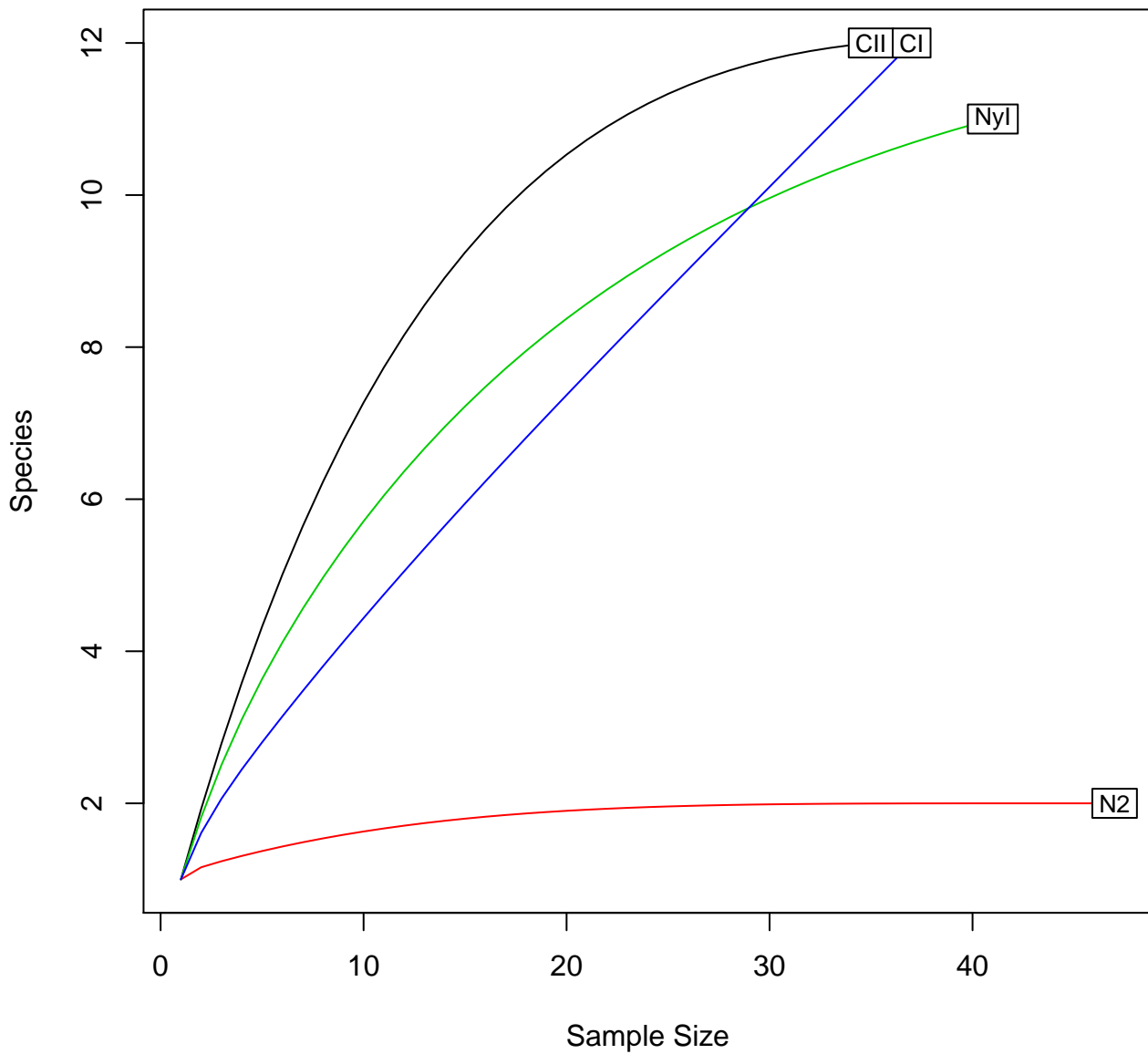

Supplement: Supplementary file 3 — Fig. S3. Rarefaction curves done with R and the VEGAN package. The number of expected species (y‐axis) is plotted as a function of the number of samples (x‐axis). Blue line: sample CI; black line: sample CII; green line: sample Ny1 and red line: sample N2. [file EMI4-7-849-s003.pdf]
